# Medical Student Balint Group – Improving Empathy and Communication; an Avenue to Relieve Burnout

**DOI:** 10.1192/bjo.2026.11336

**Published:** 2026-06-30

**Authors:** Rohaan Bukhari

**Affiliations:** Sheffield Partnership University NHS Foundation Trust, Sheffield, United Kingdom

## Abstract

**Aims:**

The Balint group was established in the 1950s as an avenue for doctors to bring forththeir experiences and feelings relating to patient interactions, in a humanistic rather than clinical way. Commonplace in Psychiatry, the use of Balint groups is spreading. Building on a previous pilot, we ran a Medical Student Balint Group weekly in a confidential, consistent space. We aimed to assess their knowledge of Balint, empathy, and communication skills and style, at the start and end of the group. This was alongside a tool for assessment of Burnout levels at the start and end of the group.

**Methods:**

A weekly Balint group cohort of 10 third year medical students was facilitated by a Core Trainee and supervised by a Consultant Medical Psychotherapist. The students were given a set of questions about the psychological factors pertaining to doctor-patient interactions, including 2 questions about their prior knowledge of these groups, and the same questionnaire was given at the end, but with 2 open-ended questions about their reflections of the process. A validated burnout self-test was also given at the start and end.

**Results:**

Weekly attendance varied slightly. All participants returned both initial questionnaires, with 8 returning both ending questionnaires. Burnout scores showed significant reduction, and students generally showed eagerness to attend Balint groups in future. On starting, half the students felt that the application of a Balint group was ‘interesting’ but afterwards most felt an expanded perspective on the doctor–patient relationship and able relate to patients better. Self-awareness and confidence with communication also improved. Qualitative results supported above quantitative results.

**Conclusion:**

Balint groups for medical students now have an increased evidence base as part of the undergraduate medical curriculum. They enhance their ability to see their patients’ perspectives, improving empathy, and markedly reducing burnout. They also enhance self-awareness and communication in medical students.

